# Change of Muscle Activity as Well as Kinematic and Kinetic Parameters during Headers after Core Muscle Fatigue

**DOI:** 10.3390/sports5010010

**Published:** 2017-01-22

**Authors:** Stephan Becker, Michael Fröhlich, Jens Kelm, Oliver Ludwig

**Affiliations:** 1 Department of Sport Science, University of Kaiserslautern, 67663 Kaiserslautern, Germany; michael.froehlich@sowi.uni-kl.de; 2 Chirurgisch-Orthopädisches Zentrum, 66557 Illingen, Germany; jens.kelm@chirurgie-illingen.de; 3 Institute of Sports Science, Saarland University, 66123 Saarbruecken, Germany; oliver.ludwig1@uni-saarland.de

**Keywords:** soccer, heading, fatigue, kinematics, kinetics, electromyography, concussion

## Abstract

In soccer, headers are a tactical measure and influenced by numerous factors. The goal of this study was to identify whether changes in kinematics and muscular activity, especially of the head-stabilizing muscles, occur during headers when the core musculature is fatigued. In two subgroups, muscular activity (12 amateur players, age 23.6 ± 4.2 years) and kinematics and dynamics (29 amateur players, age 23.7 ± 2.8 years) were examined during straight headers on a pendulum header. Data were collected before and after the core muscles were fatigued by an exercise program. Telemetric surface EMG, 3D acceleration sensor, force plate, and video recordings were used. Under fatigue, the activity of M. erector spinae and M. rectus abdominis was significantly reduced in the preparation phase of the header. The activity of M. sternocleidomastoideus was significantly increased during the jump phase, and the hip extension angle during maximum arched body tension was significantly reduced under fatigue. Jumping height, acceleration force impulse, and linear head acceleration were also significantly reduced. We conclude that fatigue of the core muscles affects the motion technique of the header and the activity of the muscle groups stabilizing the head. Therefore, the necessity of specific training in soccer should be emphasized from a medical-preventive point of view.

## 1. Introduction

Soccer is still the most popular sport in the world [1]. Men and women of all ages with different levels of expertise perform this sport [2]. The purposeful use of the head to hit the ball makes it unique in comparison to other sports [3].

Heading is subject to many influencing factors. Besides factors such as weather conditions, ball characteristics, and ball pressure, personal tactical measures, e.g., being in a standing position, jumping, sprinting, and interacting with an opponent also affect the heading process [4,5,6]. Essentially, the motion technique, fitness, coordinative skills, and the degree of fatigue decide the success or failure of a header and the potential health risk involved [7,8].

Currently, possible long-term heading effects caused by the acceleration forces on the head and concussions are a focus of interest [5,9,10]. In the course of a 15-year professional soccer career, a player performs approximately 5250 headers in matches; training headers are not included in this calculation [9,11]. It is presumed that impacts as well as concussions suffered during soccer are similar to those occurring in boxing or American football [5,9,10].

Further research is required on a neuromuscular level. Current studies show that a muscular imbalance between neck flexors and extensors is associated with an increased acceleration of the head when performing a header [8]. Additionally, Kartal et al. found increased degenerative changes in the area of the cervical vertebrae (C2–C6) in soccer players, which can be linked to the increased strain caused by headers [12]. 

During ball contact, cervical spine and head are being stabilized by a co-contraction of the neck flexors and extensors, while the acceleration required for a header is generated by the core muscles [13]. Therefore, it may be assumed that fatigue or a low training level of the stabilizing muscle groups reduce their protective potential. Another question is whether fatigue of the motion-generating core muscles can change the muscular activity of the stabilizing neck muscles. 

Taking these questions as a starting point, this study aims to investigate the extent to which fatigue of the core-stabilizing muscles affects headers in soccer. It was hypothesized that fatigue of core muscles could change neuronal activity and kinematic and kinetic parameters during headers. In particular, it was assumed that a reduced arched body tension, caused by the fatigue of core muscles, would lead to different neuronal activity of the neck flexors and extensors. 

## 2. Materials and Methods

### 2.1. Subjects

A total of 41 amateur soccer players participated in this study. The participants were divided into two independent subgroups. Using a pendulum header, muscular activity was examined in one of them (12 players, 7th division, age 23.6 ± 4.2 years, height 181.8 ± 6.3 cm, weight 74.7 ± 7.2 kg), and kinematics and dynamics in the other (29 players, 5th, 6th and 7th division, age 23.7 ± 2.8 years, height 182.4 ± 5.4 cm, weight 79.8 ± 6.8 kg). All subjects have had at least thirteen years of playing and header experience and an average training frequency of four training sessions (including matches) per week. Exclusion criteria were as follows: acute and chronic problems in the cervical spine, a concussion within the last 8 weeks, acute injury, acute infection, and illness. Goalkeepers were also excluded. Before the start of the study, all subjects were informed about the test design, procedure, and potential risks, and provided written informed consent. Participation was voluntary and did not involve any financial remuneration. The study was based and carried out in accordance with the current guidelines of the Declaration of Helsinki [14].

### 2.2. Neuromuscular, Kinetic, and Kinematic Analysis

Neuromuscular activity (myoelectric potential) when performing headers was determined by means of telemetric surface electromyography (Noraxon, TeleMyo TG2, sampling rate 1000 Hz) in line with the SENIAM standard [15]. Core muscles (M. erector spinae pars lumbalis (ESL), pars thoracalis (EST), and M. rectus abdominis (RAB)) and neck flexors and extensors (M. sternocleidomastoideus (SCM) and M. trapezius pars descendens (TPD)) were registered using Ag/AgCl adhesive electrodes (Ambu blue, 34 mm diameter). The raw data were rectified and smoothed (RMS, 50 ms window). Subsequently, the integrated EMG (IEMG) was calculated for the time intervals from 0.5 s before the jump to the jump (preparation phase), from take-off to ball contact (jump phase), and from ball contact to 0.5 s after ball contact (landing phase).

For the kinetic analysis, a 3D accelerometer (Noraxon, Scottsdale, AZ, USA) was attached to the occipital area, and a foot pressure sensor was fixed under the left forefoot (FSR, Conrad Electronic, Hirschau, Germany). They were synchronous with the EMG. Accelerometer, foot pressure sensor, and video analysis served to identify the preparation, jump, and landing phase, as well as to determine ball speed and the time of contact with the ball. The header jumps were performed on a multi-component measurement platform (Kistler, Winterthur, Switzerland, Type 9287) in order to determine the jump height, the brake force impulse, the acceleration force impulse, and the duration of the force impulses. The calibration was completed once before pre- and post-test.

To analyze the kinematic properties, marker points (12 mm diameter) were fixed to relevant anatomical landmarks (ear, C7, trochanter major, lateral femur condyle). A GoPro Hero3+ (GoPro, San Mateo, CA, USA, silver edition, resolution: 800 × 480, 120 FPS) was used for the 2D video analysis, and the video files were analyzed with Dartfish TeamPro 5.5 (Fribourg, Switzerland). Based on the video footage, the cervical spine (CS) extension and CS flexion angle (angle between ear, C7, trochanter major) at the time of maximum arched body tension or ball contact, the hip extension angle (angle between C7, trochanter major, lateral femur condyle) at the time of maximum arched body tension, and the head translation in the sagittal plane were determined for each jump. In addition, the ball speed from the time after ball contact until 0.067 s after the header was calculated.

An automatic synchronization between the camera, telemetric surface electromyography, and the force plate was not possible. A synchronization for the neuromuscular analysis of the first subgroup using of the telemetric surface electromyography, the foot pressure sensor and the 3D accelerometer was provided by Noraxon (TeleMyo TG2, Scottsdale, AZ, USA). The kinetic and kinematic analysis in the second subgroup did not require synchronization.

### 2.3. The Header

The header was performed using a header pendulum (Derbystar, model Swing, size 5, diameter 22 cm) fixed to a frame above the subject. The ball pendulum height was standardized to the distance of one ball diameter between head and pendulum. The subjects’ starting position was selected so that the ball was at the height of the forehead. The subjects were instructed to perform a header from a standing position by jumping with both legs and heading the ball as forcefully as possible in a horizontal forward direction. Three headers each were recorded for the pre- and post-test. The rest interval between trials was limited to a maximum of one minute. Three headers were analyzed in order to find a pre- and post-test with the same acceleration outcome to evaluate compensation strategies, allowing the subjects to achieve the same ball acceleration regardless of core muscle fatigue. 

### 2.4. Fatiguing Treatment

The influence of a tired or rather weak core musculature for a proper header was evaluated with five typical workout exercises for soccer players, which would not lead to a fatigue of the neck flexors and extensors.

To exhaust the core-stabilizing musculature, the subjects carried out three abdominal (dynamic leg raising, sit-ups with dynamic rolling off, and static forearm push-ups) and two dorsal exercises (dynamic trunk extension and static trunk extension) up to the point of subjective complete fatigue. All exercises were performed in one set with a one-minute break in between them. The post-test was conducted one minute after the treatment. The protocol took 15–20 minutes on average, depending on the individual physical shape of each player.

### 2.5. Statistics

The data here are expressed as mean values ± standard deviation. To determine pre-post effects, the *t*-test for dependent samples was applied. The normal distribution of the data was verified by means of the Kolmogorov–Smirnov test. The significance level was set to *p* < 0.05 and is stated exactly. Effect sizes (Cohen’s *d*) were also calculated, and values of 0.20, 0.50, and above 0.80 were considered small, medium, and large, respectively [16]. The statistical evaluation was executed using IBM SPSS (SPSS Version 20.0 for Windows, Chicago, IL, USA).

## 3. Results

In the preparation phase of the header, full fatigue of the core muscles led to reduced activity (IEMG) of M. erector spinae pars lumbalis (ESL) (T = −3.006; *p* = 0.013; *d* = −0.54), M. erector spinae pars thoracalis (EST) (T = −2.287; *p* = 0.045; *d* = −0.40), and M. rectus abdominis (RAB) (T = −2.480; *p* = 0.033; *d* = −0.26) between pre- and post-test. During the jump phase, M. sternocleidomastoideus (SCM) exhibited increased activity (T = 2.776; *p* = 0.018; *d* = 0.30). M. erector spinae pars thoracalis (EST) showed a lower level of activation (T = −2.937; *p* = 0.015; *d* = −0.54) during the landing phase. 

In kinetics between pre- and post-test, head acceleration (T = 2.081; *p* = 0.047; *d* = 0.40), acceleration force impulse (T = 2.139; *p* = 0.042; *d* = 0.23), and jump height (T = 2.105; *p* = 0.045; *d* = 0.36) were all reduced. 

In kinematics between pre- and post-test, the maximum hip extension angle (T = 3.751; *p* = 0.001; *d* = 0.51) was reduced (Table 1). 

The kinematic analysis showed qualitative changes of the movement pattern after core muscle fatigue, which we interpret as motor adaptation strategies.

**Figure 1:**
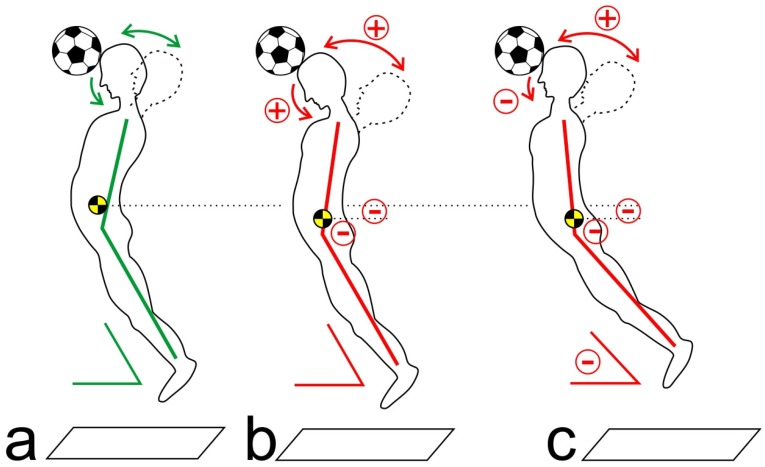
Schematic illustration of the kinematic strategies to compensate for fatigued core muscles (**a**) before fatigue; (**b**) applying Strategy 1; (**c**) applying Strategy 2 (see text).

After fatigue of the core muscles, two compensation strategies were observed during header performance. By Strategy 1, the arched body tension was reduced (smaller hip extension angle), while the counter movement of the CS (extension in the CS) and the nod motion of the head (flexion in the atlantoaxial joint) increased. Thus, the subject compensated for the reduced arched body tension by means of increased movement amplitude of the CS. In this way, the ball speed remained consistent despite the reduced movement of the trunk (Figure 1b).

When applying Strategy 2, the subjects also exhibited reduced arched body tension (in particular reduced hip extension) in the post-test. The counter movement of the CS increased, while the nod motion diminished (increased CS flexion). This means that the body adapted to the fatigued core muscles by modifying the jump direction and the alignment of the upper body. In these cases, the subjects either shifted their body’s center of gravity forward, or they changed the jump direction toward the ball (Figure 1c). 

## 4. Discussion

**Figure 2:**
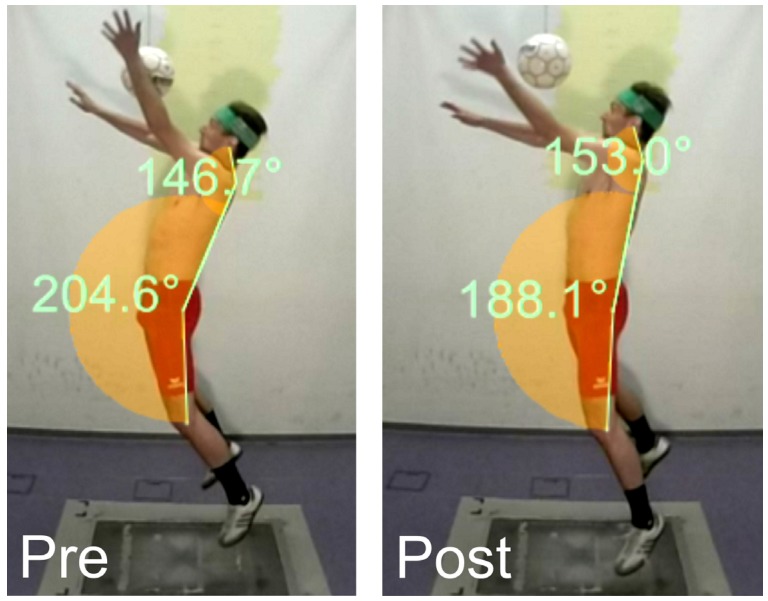
Maximum arched body tension during the pre- and post-tests (max. hip extension reduced, CS extension increased).

The subtests before and after core muscle fatigue had the same outcome, that is, an identical acceleration of the ball. Therefore, the question arises as to how the same acceleration was generated through different kinematic and muscular mechanisms, respectively. In the header preparation phase, the trunk extensors established a strained arching body position, which enabled an explosive acceleration of the upper body toward the ball by activating the trunk flexors during the jump phase [17]. However, the muscles required for the pretension (EST; ESL) were less active after fatigue, just like the trunk flexors (RAB). In terms of kinematics, this was realized in a hip extension angle reduced by an average of 3.8° (Figure 2). The acceleration force impulse was also noticeably reduced after fatigue by 4.5 Ns, and the jump height by 1.3 cm. The reason for this may be seen in fatigue-related, reduced explosive strength and a lower degree of neuromuscular coupling of the sub-segments involved. This result is in concordance with the studies on fatigue after a one-legged jump or a counter-movement jump [18,19].

The reduction of the arched body tension after the treatment can be explained by the reduced activity of EST and ESL, both of which are responsible for building up the arching body position. It remains to be seen which compensation mechanisms are applied for the missing pretension, which enable the subjects to generate a comparable output (ball speed). The assumption that the CS executes an increased extension during the counter movement and a stronger flexion at the point of ball contact in order to compensate for the fatigued core muscles was not confirmed. 

The question arises as to whether the CS applies an increased movement speed instead of a larger movement radius to compensate for the arched body tension reduced by core muscle fatigue. This means that flexion speed should be evaluated in more detail in future research. Future studies may also want to analyze (e.g., by using high-speed video footage) whether a longer ball contact transfers a greater impulse to the ball.

In the landing phase, EST activity after fatigue was significantly reduced. The trunk extensors safeguard the trunk in this phase against uncontrolled forward movement and thus ensure quick motoric return to the game. Future studies may want to examine the accuracy of the balance regulation and of other movements after landing.

This study shows that complex changes occur in the motion sequence when the core musculature is fatigued. Various motoric strategies are responsible for these changes and are presumably based on the experience and learning processes of the player. The extent to which the individual training level and the player’s position in the game have any impact on the general header strategy remains to be explained. There is only insufficient research on the smaller muscle groups around the cervical spine and their stabilizing and protective function for the sensitive cervical structures during headers. As targeted fatigue of the large core muscles leads to changed activity of the neck flexors and extensors, a potentially negative impact on the stabilization of the CS must be considered. We recommend this as a subject of further research.

The limitations of this study can be seen in the two independent subgroups and the two-dimensional analysis as well as in the amateur status of the players. Future studies may want to collect three-dimensional data with a bigger sample size and professional soccer players. Furthermore, the fatiguing treatment should involve methods of creating objective criteria of fatigued muscles. Additionally, the movement of the arms plays an important role and should undergo a quantitative analysis in the future.

## 5. Conclusions

The fatigue of the core muscles has demonstrable effects on the quality of a header’s motoric performance. The kinematic changes observed and the electromyographically measured increased activity of the SCM support the assumption that the stabilizing protection of the CS decreases in the post-test. Therefore, in both the long and the short term, athletes may be subject to an increased risk of overload injuries caused by headers [7,20]. In order to counteract changes in the kinetics and kinematics of the header when the core muscles are fatigued, we recommend to coaches and athletes that core muscles be strengthened; additionally, the cervical and neck muscles should be a regular focus of accompanying exercise programs.

## Figures and Tables

**Table 1:** Statistical overview of kinetic, kinematic, and EMG parameters.

Parameter	Pre ± SD	Post ± SD	T	*Degree of Freedom*	*p*	*d*
SCM ^1^, preparation phase	116.8 ± 56.7	109.1 ± 46.4	−0.457	10	0.657	−0.15
TPD ^2^, preparation phase	155.2 ± 50.1	156.2 ± 48.1	−0.132	10	0.898	0.02
EST ^3^, preparation phase	89.9 ± 35.4	106.4 ± 48.0	−2.287	10	0.045	−0.40
ESL ^4^, preparation phase	95.0 ± 53.2	131.3 ± 78.5	−3.006	10	0.013	−0.54
RAB ^5^, preparation phase	41.0 ± 29.72	49.1 ± 32.7	−2.480	10	0.033	−0.26
SCM, jump phase	135.3 ± 48.7	120.5 ± 50.1	2.776	11	0.018	0.30
TPD, jump phase	100.3 ± 56.6	80.1 ± 46.8	1.505	11	0.161	0.39
RAB, jump phase	192.7 ± 93.6	199.9 ± 100.8	0.036	11	0.972	−0.07
TPD, landing phase	113.1 ± 80.9	88.1 ± 35.6	1.320	10	0.216	0.40
EST, landing phase	67.6 ± 52.7	99.8 ± 66.2	−2.937	10	0.015	−0.54
ESL, landing phase	65.6 ± 37.9	223.5 ± 344.7	−1.512	10	0.161	−0.64
Acceleration of the head (G)	2.7 ± 0.5	2.5 ± 0.5	2.087	28	0.047	0.40
Acceleration force impulse (Ns)	188.5 ± 20.7	184.0 ± 19.2	2.139	26	0.042	0.23
Jump height (cm)	27.9 ± 3.6	26.6 ± 3.7	2.105	26	0.045	0.36
Max. hip extension angle (°)	214.8 ± 7.5	210.4 ± 9.6	3.751	27	0.001	0.51
Max. CS ^6^ extension angle (°)	145.5 ± 10.5	148.5 ± 9.2	−1.934	27	0.064	−0.30
CS flexion angle at ball contact (°)	131.6 ± 8.6	131.4 ± 7.5	0.090	27	0.929	0.03
Head translation (cm)	0.12 ± 0.02	0.12 ± 0.02	0.313	27	0.757	0.00

^1^ SCM = M. sternocleidomastoideus; ^2^ TPD = M. trapezius pars descendens; ^3^ EST = M. erector spinae pars thoracalis; ^4^ ESL = M. erector spinae pars lumbalis; ^5^ RAB = M. rectus abdominis; ^6^ CS = cervical spine.
